# Anticancer Activity of Novel Plant Extracts and Compounds from *Adenosma bracteosum* (Bonati) in Human Lung and Liver Cancer Cells

**DOI:** 10.3390/molecules25122912

**Published:** 2020-06-24

**Authors:** Ngoc Hong Nguyen, Qui Thanh Hoai Ta, Quang Thang Pham, Thi Ngoc Han Luong, Van Trung Phung, Thuc-Huy Duong, Van Giau Vo

**Affiliations:** 1 CirTech Institute, Ho Chi Minh City University of Technology (HUTECH), Ho Chi Minh City 700000, Vietnam; nn.hong@hutech.edu.vn; 2 Institute of Research and Development, Duy Tan University, Danang 550000, Vietnam; tathoaiqui@duytan.edu.vn; 3 Institute of Applied Science, Ho Chi Minh City University of Technology (HUTECH), Ho Chi Minh City 700000, Vietnam; pquangthang1@gmail.com (Q.T.P.); ngochanlt96@gmail.com (T.N.H.L.); 4 Center for Research and Technology Transfer, Vietnam Academy of Science and Technology, Hanoi 100000, Vietnam; pvtrung@ict.vast.vn; 5 Department of Organic Chemistry, University of Education, Ho Chi Minh City 700000, Vietnam; huydt@hcmue.edu.vn; 6 Bionanotechnology Research Group, Ton Duc Thang University, Ho Chi Minh City 700000, Vietnam; 7 Faculty of Pharmacy, Ton Duc Thang University, Ho Chi Minh City 700000, Vietnam

**Keywords:** *Adenosma bracteosum*, extract, anti-cancer, cell line, isolated compounds, caspase-3

## Abstract

Cancer is the second leading cause of death globally, and despite the advances in drug development, it is still necessary to develop new plant-derived medicines. Compared with using conventional chemical drugs to decrease the side effects induced by chemotherapy, natural herbal medicines have many advantages. The present study aimed to discover the potential cytotoxicity of ethanol extract and its derived fractions (chloroform, ethyl acetate, butanol, and aqueous) of *Adenosma bracteosum* Bonati. (*A. bracteosum*) on human large cell lung carcinoma (NCI-H460) and hepatocellular carcinoma (HepG2). Among these fractions, the chloroform showed significant activity in the inhibition of proliferation of both cancerous cells because of the presence of bioactive compounds including xanthomicrol, 5,4’-dihydroxy-6,7,8,3’-tetramethoxyflavone, and ursolic acid which were clearly revealed by nuclear magnetic resonance spectroscopy (^1^H-NMR, ^13^C-NMR, Heteronuclear Multiple Bond Coherence, and Heteronuclear Single Quantum Coherence Spectroscopy) analyses. According to the radical scavenging capacity, the 5,4’-dihydroxy-6,7,8,3’-tetramethoxyflavone compound (AB2) exhibited the highest anticancer activity on both NCI-H460 and HepG2 with IC_50_ values of 4.57 ± 0.32 and 5.67 ± 0.09 µg/mL respectively, followed by the ursolic acid with the lower percent inhibition at 13.05 ± 0.55 and 10.00 ± 0.16 µg/mL, respectively (*p* < 0.05). Remarkably, the AB2 compound induced to significant increase in the production of reactive oxygen species accompanied by attenuation of mitochondrial membrane potential, thus inducing the activation of caspase-3 activity in both human lung and liver cancer cells. These results suggest that *A. bracteosum* is a promising source of useful natural products and AB2 offers opportunities to develop the novel anticancer drugs.

## 1. Introduction

A major problem of public health, cancer is one of the main causes of death globally. The prevalence of this disease is rising, however, more rapidly in Africa, Asia, and Central and South America that make up about 70% of cancer deaths in the world [1]. Many studies have been focusing on the development of agent for cancer therapies [2,3,4]. The chemotherapy is one of the ways to treat this disease and the advances in anticancer drugs have improved patient care. Unfortunately, the conventional chemical drugs also cause adverse side effects on normal cells/tissue, such as bone marrow function inhibition, nausea, vomiting, and alopecia [5,6]. On the other hand, natural antioxidants and many phytochemicals have been recently suggested as anti-cancer adjuvant therapies because of their anti-proliferative and pro-apoptotic properties [3,4]. Hence, the continuing search for anticancer agents/compounds from plants played a critical role to find the possible ways to have safe and to decrease the side effects induced by chemotherapy since natural herbal medicines have many advantages [7,8,9,10].

Over several decades, around 200 new chemical compounds have been approved to fight cancer, 50% of that come from structurally originally natural products and their modifications to be safe and have many advantages [11,12]. Owing to their structural diversity, organic molecules (e.g., terpenes, flavonoids, alkaloids, lignans, saponins, vitamins, glycosides, oils, and other secondary metabolites) play a vital role in selective inhibition of proliferation and induction of cancerous cell death [13,14]. Among methoxylated flavones, xanthomicrol was first identified [15] and isolated from *Dracocephalum kotschyii* Boiss [16] which was able to inhibit proliferation of a number of malignant cells [16,17] because of its inhibition of endothelial cell proliferation via decreased vascular endothelial growth factor activity [18]. In terms of ursolic acid’s anti-cancer effect, many studies reported that the underlying mechanisms were the inhibition of tumorigenesis and cancer cell proliferation, as well as apoptosis modulation, cell cycle arrest prevention, and autophagy promotion through in vitro and in vivo models [19,20,21,22,23]. As part of the continuing investigation on natural antitumor from herbs, Bai et al. 2010 first isolated 5,4’-dihydroxy-6,7,8,3’-tetramethoxyflavone from the aerial parts of *Rabdosia rubescens*, which was able to exhibit cytotoxicity in a various of cancer cell lines, however, the exact mechanisms of its health benefits are unclear [24].

*Adenosma bracteosum* Bonati (*A. bracteosum*) belongs to the family Scrophulariaceae, and is used in the treatment of liver diseases because it contains around twelve compounds of essential oil such as thymol (25.6%), linalool (13.1%), and (E)-β-farnesene (9.5%), etc., [25,26]. Although this plant is reported as an efficient medicinal plant, the cytotoxicity against lung and liver cancers and the response of cell lines to the plant extract have not been described. The present study aimed to discover cytotoxic and apoptotic potential of ethanol extract and its derived fractions (chloroform, ethyl acetate, butanol, and aqueous) as well as isolated compounds of *A. bracteosum* on human large cell lung carcinoma (NCI-H460) and hepatocellular carcinoma (HepG2).

## 2. Materials and Methods

### 2.1. Chemicals and Reagents

Camptothecin, DMSO (dimethyl sulfoxide), ethanol, FBS (Fetal bovine serum), HEPES, glucose, L-glutamine, phenol red, sodium bicarbonate, sulforhodamine-B, streptomycin were purchased from Sigma-Aldrich (St. Louis, MO, USA). n-hexane, ethyl acetate, chloroform, silicagel, methanol, trichloroacetic acid, and TLC were purchased from Merck (Darmstadt, Germany).

### 2.2. Plant Material, Extraction, and Isolation

The aerial part of *A. bracteosum* was collected in December 2018 from Ba Den Mountain, Tay Ninh province, Vietnam. A voucher specimen (No. UH-B02) was deposited in the herbarium of the CirTech Institute, Ho Chi Minh City University of Technology (Vietnam). Total of 7 kg of the shade-dried and powdered aerial part of *A. bracteosum* (7 kg) was mashed in ethanol 70% for 2 days. To obtain 800 g of crude ethanol extract, the solution was filtered, followed by removing of solvent under reduced pressure. To obtain aqueous, n-hexane, chloroform (CHCl3), ethyl acetate (EtOAc), and n-butanol fractions, these extracts were then suspended in DW and successively partitioned with the solvents, respectively. The chloroform extract (104.6 g) was applied to normal phase silica gel CC, and eluted with a gradient of *n*-hexane-EtOAc (2:8 to 0:10, *v/v*) then eluted with EtOAc-CH_3_OH (1:1, *v/v*) to give 10 fractions (**A1**–**10**). Fraction **A7** (35.5 g) was fractionated in the same manner as mentioned previously to afford eight subfractions **A7.1**-**8**. Purifying the subfraction **A7.4** (5.5 g) by CC provided seven fractions (**A7.4.1**-**7**). Fraction **A7.4.1.4** was rechromatographed by CC using the mixture of *n*-hexane-CHCl_3_ (2:8, *v/v*) as mobile phase to afford compound **AB1** (135 mg). Likewise, multiple purification of fraction **A7.4.1.5** by CC yielded compound **AB2** (172 mg) and **AB3** (127 mg).

### 2.3. Free Radical Scavenging Activity Assay

The samples were measured using the stated method for the 2,2-diphenyl-1-picrylhydrazyl (DPPH) assay [27]. A total of 50 µL of sample solutions were used to react with 2 mL of DPPH• of 6–5 M in 80% methanol. All samples were scanned at the absorbance wavelength (515 nm) after 16 min. The % of DPPH• radical inhibition was calculated using the following equation: % Inhibition = [(A_C(0)_ – A_S(t)_) / A_C(0)_)] × 100, where A_C(0)_ and A_C(t)_ observed at t = 0 and 16 min, respectively.

### 2.4. Assessment of ABTS Radical Scavenging Activity

A previous procedure [28] was used to test the radical scavenging activity of the samples. Briefly, ABTS—2,2’-azino-bis(3-ethylbenzothiazoline-6-sulfonic acid) was dissolved in water, reacted with potassium persulfate (2.45 mM) to obtain the working solution. A 1.0 mL of the working solution was mixed with 10 mL samples and measured at 734 nm after 6 min. Ascorbic acid was used as positive control.

### 2.5. Brine Shrimp Lethality Bioassay

The cytotoxic activity of the samples (the ethanol and aqueous crude extracts, chloroform, ethyl acetate, n-butanol, and aqueous fractions and the isolated compound) was determined using the Brine shrimp lethality bioassay [29,30]. Briefly, brine shrimp eggs (Artemia salina) were incubated in a vessel containing sterile artificial seawater produced by dissolving 38 g of table salt in 1 L of distilled water at 28–30 °C with good aeration (using air pump), under a continuous light condition (60 W lamp) for 48 h. After hatching, nauplii were collected with a Pasteur pipette, ten brine shrimps were moved into each well containing the seawater.

Sample stock solutions were made by dissolving 5 mg of each sample in 1 mL of DMSO. Test solutions of different concentrations (1000, 800, 400, 200, 100, 50, 25, 12.5, and 6.25 μg/mL) were collected using the serial dilution technique with seawater. The solutions were transferred to individual vials and 5 mL of the seawater including 10 nauplii shrimp were taken into each vial. A control group comprising same volumes of DMSO (as in the sample vials) and 10 nauplii in 5 mL of artificial seawater was used. After 24 h, the vials were examined with a magnifying glass and the amount of nauplii survived in each vial was counted. The percent mortality of the brine shrimp nauplii was determined for control and increasing concentration from the data and the values of the LC_50_ were determined. Potassium dichromate was used as a reference standard.

### 2.6. Cell Culture and Sulforhodamine B Assay

The NCI-H460 and HepG2 were grown in Dulbecco’s Modified Eagle Medium (DMEM) containing (1% penicillin/streptomycin, 1% L-glutamine, and 10% FBS) and were incubated consecutively in culture flasks (37 °C and 5% CO_2_). The sulforhodamine B (SRB) assay was performed to determine the cytotoxicity effect of the extract, fractions and compounds as previously described [31,32]. Briefly, the cells at 7.5 × 10^3^ cells/mL were seeded in a transparent 96-well plate (Falcon, Franklin, NJ, USA) and incubated (37 °C and 5% CO_2_) for 24 h. Then, the old DMEM medium was replaced by 100 µL of the crude extract and its fractions or camptothecin at different concentrations (100, 75, 50, 30, 20, 10, 5, 2.5, 1.0, and 0.5 µg/mL), followed by incubation at 37 °C for 48 h. Subsequently, the treated and non-treated cells were fixed by adding 50 µL of cold trichloroacetic acid 50% and incubated for 1 h at 4 °C. The plates were washed with distilled water, air dried, and stained with 0.2% SRB (20–30 min, room temperature). The plates were then washed with 1% acetic acid to remove unbound dye. After air-drying, 200 µL Tris-base 10 mM was added to each well. The plates were shaken in ELISA photometer for 20 min and absorbance was measured at wavelength of 492 nm and a reference wavelength of 620 nm. The effect on the cell growth was calculated as:

I% = 1 − [OD (492 − 620) sample / OD (492 − 620) blank]) × 100%

where I% = % growth inhibition; OD = optical density. In addition, the IC_50_ values (the concentration corresponding to 50% cell-growth-inhibition rate) were also determined on the basis of linear-regression analyses. 

### 2.7. DNA Fragmentation and Apoptosis Induced by AB2

In order to analyze DNA fragmentation, NCI-H460 and HepG2 cells were induced apoptosis by treating with AB2 at IC_50_ and 2 × IC_50_. DNA purification kit was applied to extract DNA, according to the manufacturer’s instructions (Thermo Fisher Scientific, CA, USA). Passing quantification, 2 μg of each DNA sample was loaded to electrophoresis on a 1.5% agarose gel, then the gel was photographed under ultraviolet illumination after staining with ethidium bromide (10 μg/mL).

The effect of AB2 on cell apoptosis was evaluated by flow cytometer with Annexin V-FITC/PI staining kit (Thermo Fisher Scientific, CA, USA), according to the manufacturer’s instructions. Briefly, the cells were treated with AB2 compound at IC50 and 2 × IC50 concentrations for 24 h. After harvesting, cells were suspended in 300 μL binding buffer, and then stained with Annexin-FITC and/or propium iodide. Positive controls for apoptosis were stained with only Annexin-FITC. Positive controls for necrosis were stained with only propium iodide. At least 10^4^ cells were analyzed by flow cytometer (BD, Biosciences, San Jose, CA, USA) and data were analyzed using FlowJo vX.0.7 (Tree Star, Inc., Ashland, OR, USA).

### 2.8. Detection of Mitochondrial Membrane Potential

The changes in mitochondrial membrane potential were determined using TMRE (tetramethylrhodamine, ethyl ester) mitochondrial membrane potential assay kit (Abcam, Cambridge, UK), according to the manufacturer’s instructions. Briefly, NCI-H460 and HepG2 cells in 96-well plate at 1 × 10^4^ cells per well were incubated for 24 h. Cells were treated with AB2 compound at IC_50_ and 2 × IC_50_ concentrations for 24 h. After incubation time periods, the cells were harvested, washed twice in PBS, re-suspended in media supplemented with TMRE (200 nM), incubated at 37 °C for 20 min in the dark. Then, the media was replaced once with 100 µL of PBS/0.2% BSA, and then the fluorescence of TMRE was measured at an excitation wavelength of 549 nm by using a microplate reader (Perkin Elmer, Victor X5, Norwalk, CT, USA). The carbonyl cyanide-p-trifluoromethoxyphenylhydrazone (FCCP)-treated cells were used as a positive control.

### 2.9. Fluorescent Assays for Measuring Caspase-3 Activity and Intracellular Reactive Oxygen Species (ROS) Generation

Caspase activity assays in multi-well plate formats represent powerful tools for understanding experimental modulation of the apoptotic response. Caspase-3, -8, -9 activities were measured using the activity assay kit (Abcam, Cambridge, UK)) were purchased by Abcam according to the manufacturer’s guidelines. Briefly, NCI-H460 and HepG2 cells were cultured into 96-well plates at a density of 2 × 10^4^ cells/well. Then, old media were replaced by fresh ones having the AB2 compound at IC_50_ and 2 × IC_50_ concentrations, and cells were further incubated for 6 h. Total of 100 μL of Caspase reagent was added to each well. The fluorescence of each well was measured at excitation/emission (Ex/Em) = 535/620 nm, Ex/Em = 490/525 nm, and Ex/Em = 370/450 nm for Caspase 3, Caspase 8, and Caspase 9 respectively for detecting fluorescence intensity using a plate-reading fluorescence reader (Perkin Elmer, Victor X5, Norwalk, CT, USA).

ROS generation was tested by using the ROS assay kit (ab113851, Abcam) according to the manufacturer’s instructions. Briefly, the cells (5 × 10^4^ cells/well) were cultured in 96-well plates. DCFH-DA was added to the cells at 37 °C for 1 h in the dark. After incubation with AB2 compound at IC_50_ and 2 × IC_50_ concentrations for 0, 4, 8, 16, 20, and 24 h, cells were rinsed with PBS. Cells were measured on a fluorescent plate reader (Perkin Elmer, Victor X5, Norwalk, CT, USA), and mean ± standard deviation was plotted for three replicates from each condition. Tert-butyl hydrogen peroxide (TBHP), which mimics ROS activity to oxidize DCFDA to fluorescent DCF, was used as positive control.

### 2.10. Western Blot Analysis

NCI-H460 and HepG2 cells were treated with AB2 compound at IC_50_ and 2 × IC_50_ concentrations and Camptothecin (4 µg/mL), then total protein was extracted using radioimmunoprecipitation assay buffer. Following by centrifuging at 14,000 rpm for 20 min at 4 °C, total proteins were obtained, measured, and subjected onto 12% sodium dodecyl sulfate-polyacrylamide gel electrophoresis (SDS-PAGE), separated and transferred onto a nitrocellulose membrane. Then, 5% bovine serum albumin (BSA) was applied to block the membranes, followed by probing with anti-active caspase-3 and anti-β-actin primary antibodies with gentle agitation overnight at 4 °C. Afterward, the corresponding HRP-conjugated secondary antibody was applied and incubated for 1 h at room temperature after washing three times with TBST buffer (Tris-buffered saline, 0.1% Tween 20). The blots were visualized with enhanced chemiluminescence detection and quantified by densitometry using Image J version 1.47.

### 2.11. Statistical Analysis

The experiments were performed in triplicate. The data are expressed as mean ± standard deviation. Significant differences between groups were determined by using Student *t*-test. *p* value of less than 0.05 was considered significant.

## 3. Results and Discussion

### 3.1. Brine Shrimp Lethality Test

**Figure 1:**
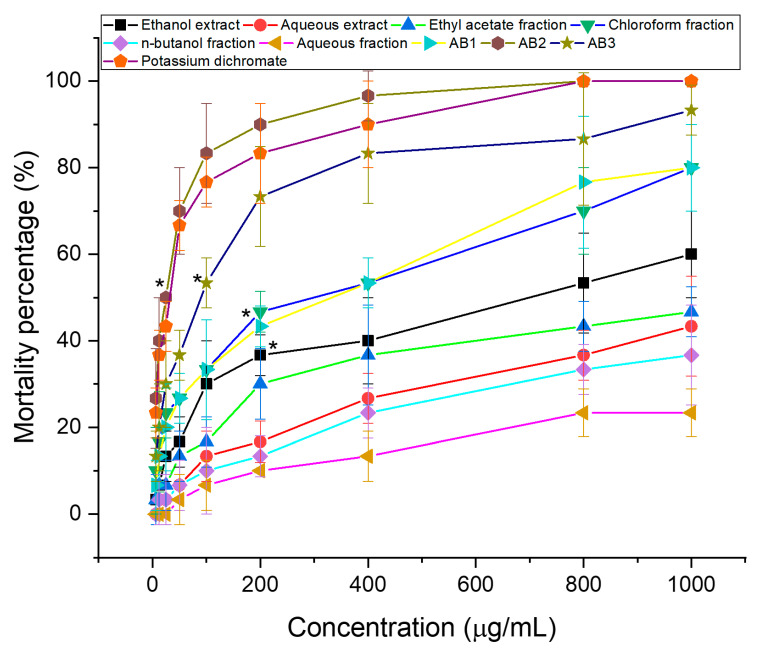
Brine shrimp lethality of extracts, fractions (A), and isolated compounds (B) at 24 h, * mean significant difference (*p* < 0.05) compared to control.

The brine shrimp assay is significantly associated with in vitro growth inhibition of human solid tumor cell lines which was demonstrated by the National Cancer Institute (NCI, USA) and it can show the value of this bioassay as a pre-screening tool for antitumor drug research [33]. This test is well correlated with antitumor activity (cytotoxicity) and can be used to monitor the activity of bioactive natural products [34]. As shown in Figure 1, the cytotoxicity demonstrates a relationship between the concentration of the samples and the degree of lethality, suggesting that the samples are biologically active. LC_50_ values were calculated using graph extrapolation as are shown in Table 1. Comparison to aqueous extract, the LC_50_ values for the ethanol extract at 24 h were 647.64 μg/mL, has revealed that it more exhibited toxic expressions (LC_50_ was less than 1.0 mg/mL) against the brine shrimp [35]. The ethanol extract was then used to guide the fractionation process of the plant extract to isolate potential anti-cancer compounds. The chloroform fraction from ethanol extract was the most active among all fraction. Ethyl acetate fraction, n-butanol fraction, and aqueous fraction gave LC_50_ > 1000 μg/mL, that are considered to be inactive. The variation in their results is possibly because of the different polarities of the solvents, the ethanol solvent is less polar than aqueous one and the phytochemicals of the ethanol extract contain specific molecules that have provided its cytotoxic activity against brine shrimp. As a result, the chloroform fraction of ethanol extract exhibited more toxicity against the brine shrimp at 205.58 μg/mL compared to that of other fractions, which was further used to isolate compounds.

### 3.2. In Vitro Screening for Cytotoxic Activity of Extracts and Fractions

**Figure 2:**
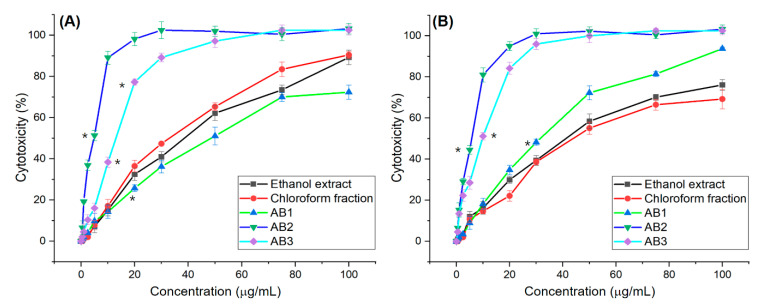
Cytotoxicity of extract, fraction, and compounds on NCI-H460 (**A**) and HepG2 (**B**) cells at different concentration, * mean significant difference (*p* < 0.05) compared to control.

The cytotoxic effect of extracts and fractions from *A. bracteosum* on the growth of NCI-H460 and HepG2 cancer cell lines were investigated by SRB assay. The cell inhibition activity of ethanol and aqueous extracts, chloroform, ethyl acetate, *n*-butanol, and aqueous fractions of *A. bracteosum* showed in Figure 2. The ethanol extract had high activities on the human non-small cell lung cancer carcinoma NCI-H460 (90.46 ± 1.61%) and HepG2 (69.23 ± 4.71%) cell lines but at the same concentration of 100 μg/mL, aqueous had low activities (<25%). The chloroform fraction from ethanol extract had the highest activity as compared to other fractions that percentage of cytotoxicity on cell line NCI-H460 and HepG2 was 89.29 ± 0.63% and 76.40 ± 3.62%, respectively. As revealed in Figure 2, ethanol extract and chloroform fraction had significant cytotoxic activity against the cells in increasing dose concentration. The death of 50% of the tumor cells of ethanol extract on HepG2 and NCI-H460 was 39.15 ± 0.61 and 30.31 ± 1.60 μg/mL while IC_50_ of chloroform fraction was 36.34 ± 0.48 and 38.35 ± 2.04, respectively. HepG2 cell was more sensitive to chloroform fraction than ethanol extract. The chloroform fraction was therefore further studied for the isolation of pure compounds.

### 3.3. Spectroscopic Data of Isolated Compounds

The NMR spectra were measured on Bruker 500 Avance spectrometer (Karlsruhe, Germany) (500 MHz for ^1^H NMR and 125 MHz for ^13^C NMR) spectrometers with tetramethylsilane (TMS) as internal standard. Chemical shifts are expressed in ppm with reference to the residual protonated solvent signals (DMSO-*d_6_* with *δ*_H_ 2.50 and δ_C_ 39.52, acetone- *d_6_* with δ_H_ 2.05 and δ_C_ 29.840). The EIMS were recorded on a HRESIMS Bruker MicroTOF (Billerica, MA, USA). TLC was carried out on precoated silica gel 60 F_254_ and spots were visualized by UV_254nm_, UV_365nm_ lamp (Spectroline, Westbury, NY, USA). Gravity column chromatography was performed with silica gel 60 (0.040–0.063 mm).

**Figure 3:**
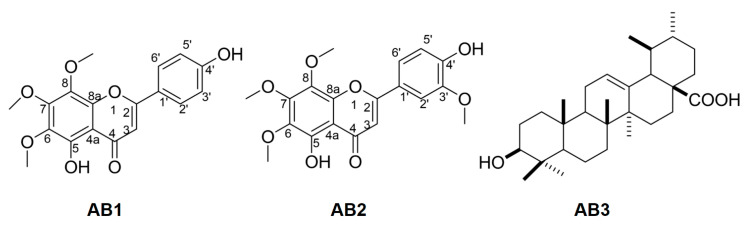
Chemical structures of AB1, AB2 and AB3.

*Compound AB1:* Yellow amorphous powder. ESI-MS *m/z*: 343.08 [M − H]^−^. ^1^H-NMR (500 MHz, CD_3_OD): δ 7.85 (1H, *d*, *J* = 8.0 Hz, H-2′/H-6′), 6.94 (1H, *d*, *J* = 8.0 Hz, H-3′/H-5′), 6.60 (1H, s, H-3), 4.09 (3H, s, OCH_3_), 3,97 (3H, s, OCH_3_), 3.90 (3H, s, OCH_3_). ^13^C-NMR (125 MHz, Acetone–d_6_): δ 183.1 (C-4), 152.0 (C-5), 164.3 (C-2), 161.3 (C-4′), 149.0 (C-8a), 136.0 (C-8), 133.0 (C-6), 128.5 (C-2′), 128.5 (C-6′), 122.3 (C-1′), 116.1 (C-3′), 116.1 (C-5′), 106.7 (C-4a), 102.8 (C-3), 61.4 (-OCH_3_), 61.0 (-OCH_3_), 60.1 (-OCH_3_). These spectroscopic data were consistent with those of xanthomicrol [16] (Figure 3). 1D and 2D NMR spectra of **AB1** were also provided in Supplementary Materials file.

**Figure 4:**
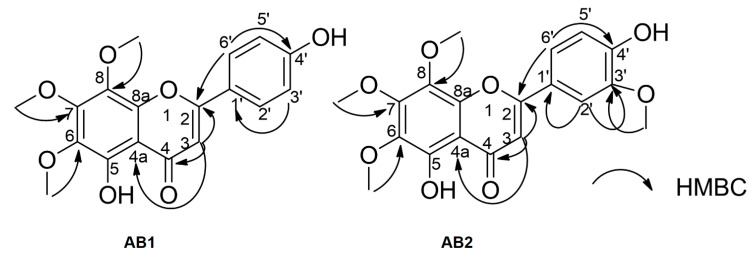
Selected heteronuclear multiple bond correlation (HMBC) correlations from **AB1** and **AB2**.

*Compound AB2:* Yellow amorphous powder. ESI-MS *m/z*: 373.10 [M-H]^-^. ^1^H-NMR (500 MHz, Acetone–d_6_): δ 7.66 (1H, *dd*, *J* = 1.5, 8.0 Hz, H-2′), 7.65 (1H, *brs*, H-6′), 7.04 (1H, *d*, *J* = 8.0 Hz, H-3′), 6.75 (1H, s, H-3), 4.09 (3H, s, OCH_3_), 3.97 (3H, s, OCH_3_), 3.90 (3H, s, OCH_3_), 3.88 (3H, s, OCH_3_). ^13^C-NMR (125 MHz, Acetone–d_6_): 183.0 (C-4), 164.4 (C-2), 152.0 (C-7), 151.0 (C-4′), 148.0 (C-8a), 145.7 (C-5′), 136.0 (C-8), 133.0 (C-6), 122.4 (C-1′), 120.6 (C-2′), 115.6 (C-3′), 109.6 (C-6′) 106.7 (C-4a), 103.0 (C-3), 61.4 (-OCH_3_), 61.0 (-OCH_3_), 60.1 (-OCH_3_), 55.6 (-OCH_3_). These results were consistent with those of 5,4’-dihydroxy-6,7,8,3’-tetramethoxyflavone [24,36,37] (Figure 3). 1D and 2D NMR spectra of **AB2** were also provided in Supporting Materials file. NMR data assignments were completed through detailed analysis of HMBC experimental data (Figure 4).

*Compound AB3:* White amorphous powder. ESI-MS *m/z:* 455.37 [M-H]^- 1^H-NMR (500 MHz, DMSO–d_6_): δ 0.66 ppm (1H, d, *J* = 11.3 Hz, H-5), 0.77 ppm (3H, s, 24-CH_3_), 0.81 ppm (3H, s, 26-CH_3_), 0.85 (3H, d, *J* = 6.4 Hz, 29-CH_3_), 0.90 ppm (3H, d, *J* = 4.2 Hz, 25-CH_3_), 0.92 (3H, s, 30-CH_3_), 0.94 (3H, d, *J* = 6.2 Hz, 23-CH_3_), 1.08 (3H, s, 27-CH_3_), 1.99 ppm (1H, dd, *J* = 4.1, 13.4 Hz, H-6), 2.11 ppm (1H, d, *J* = 13.1 Hz, H-18), 3.00 ppm (1H, dd, *J* = 6.8, 9.9 Hz, H-3), 5.14 ppm (1H, dd, *J* = 3.4, 6.8 Hz, H-12). ^13^C-NMR (125 MHz, DMSO–d_6_): δ 178.0 (C-28), 138.4 (C-13), 124.4 (C-12), 76.9 (C-3), 54.8 (C-5), 52.4 (C-18), 47.0 (C-17), 46.8 (C-9), 41.6 (C-14), 39.1 (C-8), 38.5 (C-4), 38.5 (C-19), 38.4 (C-20), 38.3 (C-1), 36.5 (C-10), 36.3 (C-22), 27.0 (C-2), 18.0 (C-6), 32.7 (C-7), 30.2 (C-21), 28.3 (C-23), 27.6 (C-15), 23.8 (C-16), 23.3 (C-27), 22.9 (C-11), 21.1 (C-30), 17.0 (C-26), 16.9 (C-29), 16.1 (C-24), 15.2 (C-25). This compound was identified as ursolic acid [19] (Figure 3) and also demonstrated to be effective for the treatment of a wide spectrum of diseases [38] including antioxidant [39], anti-inflammatory [40], antibacterial [41], antiviral [41], antifungal [41], antipyretic [38], anticancer [42], antitumor [42], antiwrinkle [43], anti-hypertension [44], and hepatoprotective activities [45].

Indeed, LC_50_ values of AB1, AB2, and AB3 were observed to be 202.80, 20.34, and 65.71 μg/mL, respectively, whereas this figure was revealed for the positive control (potassium dichromate) at 24 h was 27.75 µg/mL, indicating these toxic compounds expressions well against the brine shrimp. Hence, further investigation of these compounds to their toxic expressions on cancer cell lines should be pursued.

### 3.4. Bioactivity of Isolated Compounds

#### 3.4.1. Antioxidant Activity Assessments

**Figure 5:**
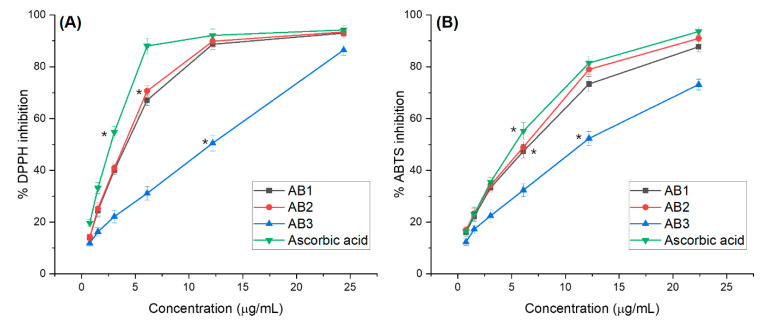
(**A**) 2,2-diphenyl-1-picrylhydrazyl (DPPH) and (**B**) ABTS+ radical scavenging activities. AB1: Xanthomicrol, AB2: 5,4’-dihydroxy-6,7,8,3’-tetramethoxyflavone, AB3: Ursolic acid, ABTS: 2,2’-azino-bis(3-ethylbenzothiazoline-6-sulfonic acid Values are given as mean ± standard deviation, * mean significant difference (*p* < 0.05) compared to control.

ABTS+ and 2,2-diphenyl-1-picrylhydrazile (DPPH) assays are widely used to evaluate compounds’ ability to determine their antioxidant potential. The free radical inhibition of three compounds and reference antioxidant (ascorbic acid) at concentrations was shown in Figure 5A, the DPPH scavenging activity of AB1, AB2, and AB3 increased progressively with the concentration. AB2 was the most active radical scavenging which showed an IC_50_ of 4.04 µg/mL and was 1.37 fold lower than that of ascorbic acid (IC_50_ = 2.95 µg/mL), followed by AB1 and AB3 with the IC_50_ values were 4.45 and 11.93 µg/mL, respectively (*p* < 0.05).

The ABTS+ assay is additionally an important method for quantifying radical scavenging activity, which can provide comparable results to those obtained in the DPPH assay. As indicated in Figure 5B, three compounds significantly exhibited ABTS-free radical scavenging activity. Three compounds had an antioxidant activity comparable to that of ascorbic acid. AB2 was a strong active radical scavenger, showing an IC_50_ of 6.53 ± 0.16 µg/mL and 1.24 times lower than that of ascorbic acid, followed by AB1 and AB3 with the IC_50_ values of 7.09 and 11.41 µg/mL, respectively (*p* < 0.05). In both the assays, AB2 exhibited the highest ABTS and DPPH radical scavenging activities. The correlation between DPPH and ABTS methods used to measure the antioxidant activity of the compounds has been examined. The DPPH radical activity also showed a strong correlation with ABTS (R^2^ = 97.56%).

Reactive oxygen species (ROS) cause a variety of cancers in humans [46]. ROS can damage macro biomolecules such as proteins, lipids, and DNA and reduce the DNA repair capability that can result in normal cells being transformed into cancer cells by mutating key genes [47]. Research on potent antioxidants or scavengers thus contributes to the prevention of cancer. Some previous studies have shown that phenols and flavonoids have strong antioxidants and are effective anticancer agents through anti-angiogenic and apoptosis activities [48,49]. In fact, in this study, A2, a flavonoid showed good antioxidant and cytotoxic effects.

#### 3.4.2. Brine Shrimp Bioassay

AB1, AB2, and AB3 were identified as confirmed by their cytotoxic effects through brine shrimp bioassay (Figure 1). LC_50_ values of AB1, AB2, and AB3 were 202.80, 20.34, and 65.71 μg/mL, respectively, which demonstrated (possessed) significant activity against the brine shrimp. The positive control LC_50_ values were 27.75 μg/mL, indicating toxic expressions against the brine shrimp. AB2 had a higher LC_50_ than the positive control, highlighting the cytotoxic effect.

### 3.5. In Vitro Screening for Cytotoxic Activity of Compounds

The cell inhibition activity of compounds including AB1, AB2, and AB3 is presented in Figure 2. Calculation of the dose of AB1, AB2, and AB3 revealed the following IC_50_ values for NCI-H460 and HepG2 cells: 32.5 ± 0.41 and 49.2 ± 0.81 µg/mL; 4.57 ± 0.32 and 5.67 ± 0.09 µg/mL; 13.05 ± 0.55 and 10.00 ± 0.16 µg/mL, respectively. According to the criteria of the National Cancer Institute and Geran protocol, extracts with IC_50_ ≤ 20 µg/mL = highly cytotoxic, IC_50_ ranged between 21 and 200 µg/mL = moderately cytotoxic, IC_50_ ranged between 201 and 500 µg/mL = weakly cytotoxic, and IC_50_ > 501 µg/mL = no cytotoxicity [50,51,52]. These results clearly reveal that test of the ethanol extract, chloroform fraction and AB1 on the two cell lines revealed less cytotoxicity. Interestingly, the compound AB2 (5,4’-dihydroxy-6,7,8,3’-tetramethoxyflavone) and AB3 (ursolic acid) have excellent cytotoxic activity for two types of cancer cells, in which the highest percent anticancer activity was observed in AB2, followed by the ursolic acid with the lower percent inhibition at the same concentration. These compounds AB2 and AB3 are greatly considered to have in vitro cytotoxic activity with an IC_50_ value ≤10 µg/mL for the cells.

Among the isolated compounds, ursolic acid (AB3) has been well documented as a naturally synthesized pentacyclic triterpenoid, widely distributed in different fruits and vegetables [53,54,55] and has been known as an excellent anticancer agent [8,11] by inducing apoptosis in several human cancer cells [56,57]. Remarkably, the compound 5,4’-dihydroxy-6,7,8,3’-tetramethoxyflavone (AB2) was isolated for the first time in the *A. bracteosum.* Further research investigating the cellular and molecular mechanisms underlying the effects of 5,4’-dihydroxy-6,7,8,3’-tetramethoxyflavone is required for the development of new therapeutic agents.

### 3.6. DNA Fragmentation and Apoptosis Induced by 5,4’-Dihydroxy-6,7,8,3’-Tetramethoxyflavone

**Figure 6:**
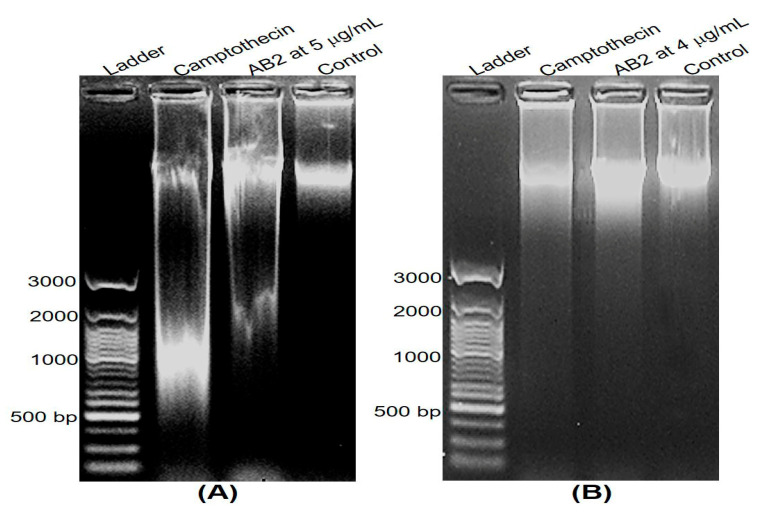
Apoptosis induced by 5,4’-dihydroxy-6,7,8,3’-tetramethoxyflavone at its IC_50_ in HepG2 (**A**) and NCI-H460 (**B**) cells.

To seek the mechanism of cell death mediated by 5,4’-dihydroxy-6,7,8,3’-tetramethoxyflavone (AB2), we first performed DNA fragmentation assay, which is characteristic for apoptosis. We treated the cells with 5,4’-dihydroxy-6,7,8,3’-tetramethoxyflavone at its IC_50_ for 24 h, and DNA was then isolated and separated by agarose gel electrophoresis. As shown in Figure 6A, the HepG2 cells treated with 5 μg/mL of AB2 showed significant fragmentation after 24 h of AB2 treatment compared to the treatment of camptothecin at 4 µg/mL. Also, an oligo nucleosomal ladder of fragmented DNA was obtained after the treatment in NCI-H460 cells (Figure 6B), whereas no DNA fragments were observed when untreated in both cells. These data strongly suggest that 5,4’-dihydroxy-6,7,8,3’-tetramethoxyflavone is a potent inducer of apoptosis in the cells.

**Figure 7:**
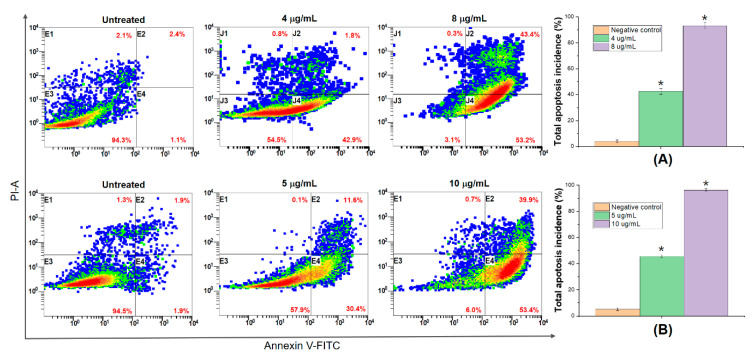
The total percentages of AB2-induced apoptosis in NCI-H460 (**A**) and HepG2 (**B**) cells, * mean significant difference (*p* < 0.05) compared to control.

In addition, to further evaluate the potential mechanism of the compound 5,4’-dihydroxy-6,7,8,3’-tetramethoxyflavone-induced inhibition of human lung and hepatocyte carcinoma cell proliferation, we continuously examined the mode of cell death on NCI-H460 and HepG2 cells Annexin V-FITC/propidium iodide (PI) staining caused by the compound. Meanwhile, PI was used to only stain necrotic cells because of its increased membrane permeability and eventual propidium uptake in the cells [58]. In this study, Figure 7 reveals total apoptosis percent from treated and untreated NCI-H460 and HepG2 cells which were determined through the flow-cytometric outcomes. As shown in Figure 7A, NCI-H460 cells were treated with AB2 at 4 µg/mL (~IC_50_) caused the lowest apoptosis (42.1%) within 24 h of treatment, relative to the control cells (~5.1%), whereas the figures seemed to be similar for HepG2 cells (Figure 7B). Remarkably, AB2 exposure, (8 µg/mL~2 × IC_50_) and (10 µg/mL~2 × IC_50_), caused a significant increase in apoptosis and necrosis in both NCI-H460 and HepG2 cells, which were about 2.3-fold and 2.2-fold higher than that of at IC_50_ doses, respectively. With respect to the cytotoxicity effect, AB2 could lead to DNA damage, apoptosis in terms of the number of cell death events, because it contains a number ROS radicals [59].

### 3.7. 5,4’-Dihydroxy-6,7,8,3’-Tetramethoxyflavone Triggers Apoptosis Sensitivity by ROS- Caspase-3 Mediated in Human Hepatocyte Carcinoma Cell

**Figure 8:**
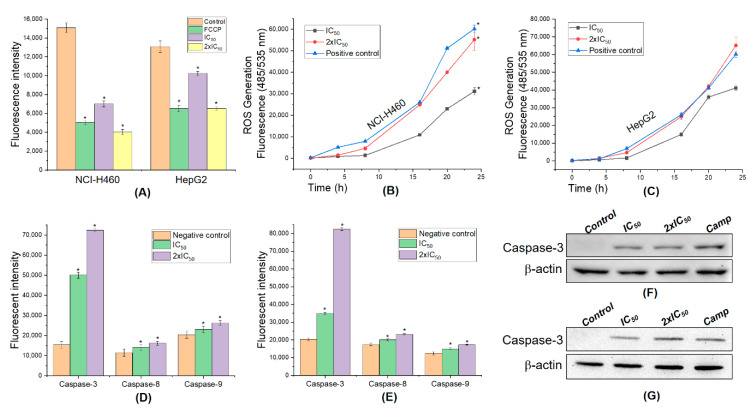
Potential mechanism of action of 5,4’-dihydroxy-6,7,8,3’-tetramethoxyflavone on human liver and lung cancer cells. (**A**) Mitochondrial membrane potential; ROS production in NCI-H460 (**B**) and HepG2 **(C**) cells by DCFH-DA essay. The caspase activity in the NCI-H460 (**D**) and HepG2 (**E**) cells treated with AB2. Expression of active caspase-3 protein on NCI-H460 (**F**) and HepG2 (**G**) cells was confirmed by Western blot analysis, * mean significant difference (*p* < 0.05) compared to control.

The disruption of mitochondrial membrane potential may result in apoptosis [60]; thus, we next evaluated, by TMRE assay, whether AB2 mediated the cells. FCCP treatment as in the positive control group caused a significant loss of mitochondrial membrane potentials in comparison to the control group (*p* < 0.05). Interestingly, the same figure was observed following the treatment (IC_50_ and 2 × IC_50_) with AB2 (*p* < 0.05), strongly revealing the presence of AB2-mediated perturbation of mitochondrial metabolic activity in NCI-H460 and HepG2 cells (Figure 8A).

ROS is known to play a dual role; either that may be harmful depending on their accumulation levels, generally contributing to cell death either by apoptosis or necrosis at the levels beyond the cellular antioxidant defense mechanisms [60,61,62]. Generally, higher mitochondrial membrane potential results in greater adenosine triphosphate (ATP) production and greater ROS production [63]. Thus, the intracellular ROS levels were determined by measuring the intensity of a highly fluorescent derivative 2′,7′-dichlorofluorescein (DCF) which is generated from an externally applied non-fluorescent substance, DCFH-DA by the cellular redox reactions. As shown in Figure 8B–C, AB2 dose-dependently increased the ratio of green fluorescence in NCI-H460 and HepG2 cells suggesting that AB2 excitants production of intracellular ROS in the cells compared to the control group, camptothecin (*p* < 0.05). The antioxidant from AB2 succeeded in inverting the cytotoxic effect of both cells. These figures strongly indicate that AB2 treatment in a concentration- and time- dependent manner led to ROS-dependent and independent cell death in human lung and hepatocellular carcinoma cells.

During apoptosis, the permeabilization of the mitochondrial outer membrane caused the release of cytochrome c [64], which induces caspase activation to orchestrate the death of the cell due to its loss of mitochondrial function and generation of ROS [65]. Overproduction of ROS could be associated with cell homeostasis imbalance, mitochondrial damage, and apoptosis [60,66]. Activation of apoptosis pathways through the mitochondrial pathway could be is one of the key steps in apoptosis [67,68], which is generally related to recruitment of caspase family proteins including caspase-8 and/or caspase-3 [69]. Thus, the caspase activities pathway is checked to evaluate whether or not AB2 affects the induction of apoptosis. As shown in Figure 8D–E, the cells treated with AB2 resulted in increased percentage of caspase-3, -8, -9 activities compared to the negative control. However, AB2 did not affect much the caspase-8 and -9 activities and resulted in slightly increased expression in the cells in comparison to caspase-3 outcomes. Indeed, there was a significantly increased apoptotic cell frequency in the cells by ~4.5 and ~7.2-fold by treatment with 5,4’-dihydroxy-6,7,8,3’-tetramethoxyflavone at IC_50_ and 2 × IC_50_ respectively, as compared with the untreated groups, suggesting that AB2 stimulates caspase-3 in human lung and hepatocellular carcinoma cells.

Finally, the expression level of apoptosis-related active protein (cleaved) caspase-3 was confirmed by Western blot assay. The expression of active caspase-3 protein was clearly revealed by only IC_50_ and 2xIC_50_ of AB2 treatment compared to the positive control group (Figure 8F–G). This figure is consistent in comparison with the significantly increased levels of active caspase-3 from fluorescence assay. Our results revealed that the role of 5,4’-dihydroxy-6,7,8,3’-tetramethoxyflavone-induced apoptosis in both human lung and hepatocellular carcinoma cells was dependent on the activation caspase-3.

These findings revealed that these extracts and compounds are very potent antioxidants because of their radical scavenging capacity and their capacity to stimulate ROS-mediated mitochondrial pathway because of the activation of caspase-3. The interactions may result from new anticancer molecules in which antioxidants can also be enhanced to eliminate cancer cells through the apoptosis pathway, where antioxidants from medicinal plants have shown a great cytotoxic potential [70,71]. This has been demonstrated through caspase-independent cell death from *H. speciosa* [72] and *J. Decurrens* [73], whereas others such as *C. adamantium* [74], *S. velutina* [72], and *S. Adstringens* [75] destroyed malignant haematologic cells or melanoma cells through apoptosis. The search for new antioxidants containing toxicity profile is desirable, and the *A. bracteosum* (Bonati) demonstrated here may represent interesting targets for this purpose.

## 4. Conclusions

The current investigation strongly demonstrate that *A. bracteosum* could significantly inhibit the growth of human NCI-H460 and HepG2 cells as well as the brine shrimp. Importantly, a new compound, 5,4’-dihydroxy-6,7,8,3’-tetramethoxyflavone was investigated, and demonstrated to be probably by ROS-mediated mitochondrial pathway, followed by activated caspase-3, associated with the apoptosis cells death. These results suggest that *A. bracteosum* is a promising source of useful natural products and the new compound offers opportunities to develop novel anticancer drug after its full apoptosis activity has been clinically addressed.

## Figures and Tables

**Table 1:** Brine shrimp toxicity expressed as LC_50_ value.

Sample	Ethanol Extract	Aqueous Extract	Chloroform Fraction	Ethyl Acetate Fraction	*n*-Butanol Fraction	Aqueous Fraction	Compound			Potassium Dichromate
							AB1	AB2	AB3	
LC_50_ (µg/mL)	647.64	>1000	205.58	>1000	>1000	>1000	202.8	20.34	65.71	27.75

## Supplementary Materials

The following are available online at https://www.mdpi.com/1420-3049/25/12/2912/s1. Figure S1: ESI mass spectrum of AB1. Figure S2: The ^1^H NMR spectrum of AB1 in methanol-*d*_4_. Figure S3: The ^13^C NMR spectrum of AB1 in acetone-*d*_6_. Figure S4: HSQC spectrum of AB1 in acetone-*d*_6_. Figure S5: HMBC spectrum of AB1 in Acetone-*d*_6_. Figure S6: EI mass spectrum of AB2. Figure S7: The ^1^H NMR spectrum of AB2 in acetone-*d*_6_. Figure S8: The ^13^C NMR spectrum of AB2 in acetone-*d*_6_. Figure S9: HSQC spectrum of AB2 in acetone-*d*_6_. Figure S10: HMBC spectrum of AB2 in Acetone-*d*_6_. Figure S11: The ^1^H NMR spectrum of AB3 in DMSO-*d*_6_. Figure S12: The ^13^C NMR spectrum of AB3 in DMSO-*d*_6_. Table S1: NMR spectral data of AB1 and AB2 compounds. Table S2: Compare AB3 compound spectral data and reference.
