# Supplementary material for: Anticancer Activity of Novel Plant Extracts and Compounds from Adenosma bracteosum (Bonati) in Human Lung and Liver Cancer Cells
Source: Molecules. 2020 Jun 24;25(12):2912. doi: 10.3390/molecules25122912 (PMC7356985; doi:10.3390/molecules25122912)
Supplement: Supplementary file 1 [file molecules-25-02912-s001.pdf]

*molecules*

*Article*

# Anticancer Activity of Novel Plant Extracts and Compounds from *Adenosma bracteosum* (Bonati) in Human Lung and Liver Cancer Cells

**Ngoc Hong Nguyen [1], Qui Thanh Hoai Ta [2], Quang Thang Pham [3], Thi Ngoc Han Luong [3], Van Trung Phung [4], Thuc-Huy Duong [5] and Van Giau Vo [6,7,*]**

1. CirTech Institute, Ho Chi Minh City University of Technology (HUTECH), Ho Chi Minh City 700000, Vietnam, nn.hong@hutech.edu.vn
2. Institute of Research and Development, Duy Tan University, Danang 550000, Vietnam, tathoaiqui@duytan.edu.vn
3. Institute of Applied Science, Ho Chi Minh City University of Technology (HUTECH), Ho Chi Minh City 700000, Vietnam, pquangthang1@gmail.com (Q.T.P.); ngochanlt96@gmail.com (T.N.H.L.)
4. Center for Research and Technology Transfer, Vietnam Academy of Science and Technology, Hanoi 100000, Vietnam, pvtrung@ict.vast.vn
5. Department of Organic Chemistry, University of Education, Ho Chi Minh City 700000, Vietnam, huydt@hcmue.edu.vn
6. Bionanotechnology Research Group, Ton Duc Thang University, Ho Chi Minh City 700000, Vietnam
7. Faculty of Pharmacy, Ton Duc Thang University, Ho Chi Minh City 700000, Vietnam

* Correspondence: vovangiau@tdtu.edu.vn

Academic Editors: José Antonio Lupiáñez, Amalia Pérez-Jiménez and Eva E. Rufino-Palomares

Received: 30 May 2020; Accepted: 16 June 2020; Published: date

# Supplement Information

**Table S1.** NMR spectral data of AB1 and AB2 compounds.

| | **AB1** | | **AB2** | |
|---|---|---|---|---|
| | 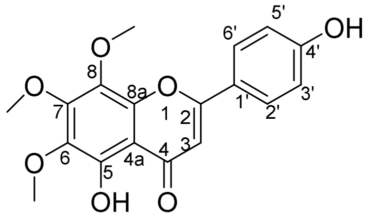 | | 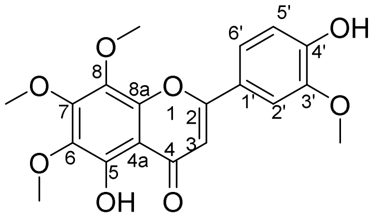 | |
| **N** | **$\delta_H$, *J (Hz)*** | **$\delta_C$** | **$\delta_H$, *J (Hz)*** | **$\delta_C$** |
| 2 | | 164.3 | | 164.4 |
| 3 | 6.60 (*s*) | 102.8 | 6.75 (*s*) | 103.0 |
| 4 | | 183.1 | | 183.0 |
| 4a | | 106.7 | | 106.7 |
| 5 | | 161.8 | | 161.8 |
| 6 | | 133.0 | | 133.0 |
| 7 | | 152.0 | | 152.0 |
| 8 | | 136.0 | | 136.0 |
| 8a | | 149.0 | | 148.0 |
| 5-OH | | | | |
| 7-$OCH_3$ | 4.09 (*s*) | 61.4 | 4.09 (*s*) | 61.4 |
| 6-$OCH_3$ | 3.97 (*s*) | 61.0 | 3.97 (*s*) | 61.0 |
| 8-$OCH_3$ | 3.90 (*s*) | 60.1 | 3.90 (*s*) | 60.1 |
| 1′ | | 122.3 | | 122.4 |

| | | | | |
|---|---|---|---|---|
| 2′ | 7.85 (*d*, *8.0*) | 128.5 | 7.66 (*dd*, *8.0, 1.5*) | 120.6 |
| 3′ | 6.94 (*d*, *8.0*) | 116.1 | 7.04 (*d*, *8.0*) | 115.6 |
| 4′ | | 161.3 | | 151.0 |
| 5′ | 6.94 (*d*, *8.0*) | 116.1 | | 145.7 |
| 6′ | 7.85 (*d*, *8.0*) | 128.5 | 7.65 (*brs*) | 109.6 |
| 3′-$OCH_3$ | | | 3.88 (*s*) | 55.6 |

**Table S2.** Compare AB3 compound spectral data and reference.

| **N (H)** | **$^{1}$H-NMR** | |
|---|---|---|
| | **AB3** | **Ursolic acid (**(Harmand *et al.*, 2003) |
| 1 | | 1.56 (2H, m) |
| 2 | | 1.43 (2H, m) |
| 3 | 3.00 (1H, td, J = 6.8; 9.9) | 3.01 (1H, dd, J = 5.2; 9.5) |
| 4 | | |
| 5 | 0.66 (1H, d, J = 11.3) | 0.66 (1H, s) |
| 6 | 1.99 (1H, dd, J = 4.1;13.4) | 1.47 (1H, m, H-6a) |
| | | 1.29 (1H, m, H-6b) |
| 7 | | 1.27 (2H, m) |
| 8 | | |
| 9 | | 1.58 (1H, s) |
| 10 | | |
| 11 | | 1.92 (2H, dd, J = 13.7; 3.5) |
| 12 | 5.14 (1H, td, J = 3.4; 6.8) | 5.14 (1H, dd, J = 13.7; 3.5) |
| 13 | | |
| 14 | | |
| 15 | | 1.01 (2H, m) |
| 16 | | 1.53 (2H, m) |
| 17 | | |

| | | |
|---|---|---|
| 18 | 2.11 (1H, d, J = 13.1) | 2.12 (1H, d; J = 11.1) |
| 19 | | 1.31 (1H, m) |
| 20 | | 1.52 (1H, m) |
| 21 | | 1.29 (2H, m) |
| 22 | | 1.54 (2H, m) |
| 23 | 0.94 (3H, d, J = 6.2) | 0.90 (3H, s) |
| 24 | 0.77 (3H, s) | 0.68 (3H, s) |
| 25 | 0.90 (3H, d, J = 4.2) | 0.87 (3H, s) |
| 26 | 0,81 (3H, s) | 0.69 (3H, s) |
| 27 | 1.08 (3H, s) | 1.05 (3H, s) |
| 28 | | |
| 29 | 0.85 (3H, d, J = 6.4) | 0.82 (3H, d, J = 5.9) |
| 30 | 0.92 (3H, s) | 0.92 (3H, d, J = 6.8) |

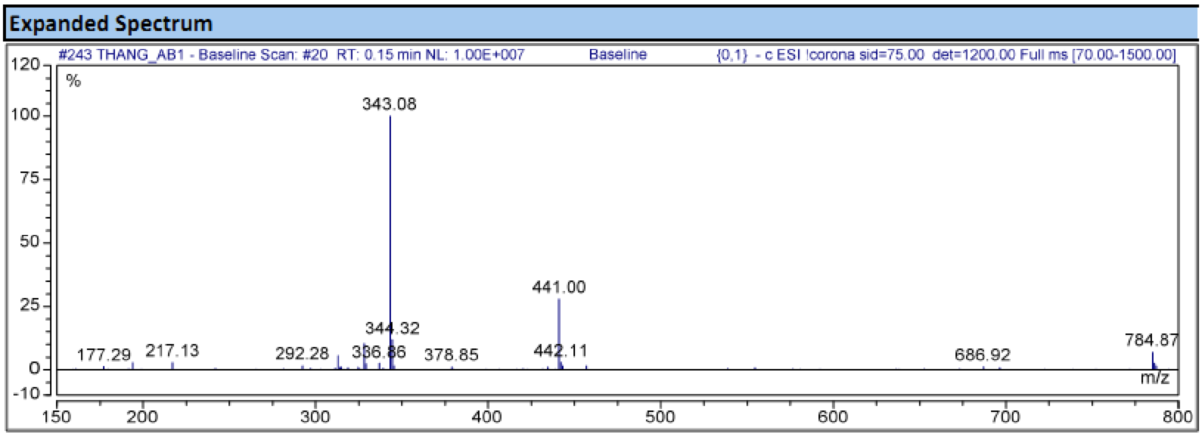


**Figure S1.** ESI mass spectrum of **AB1.**

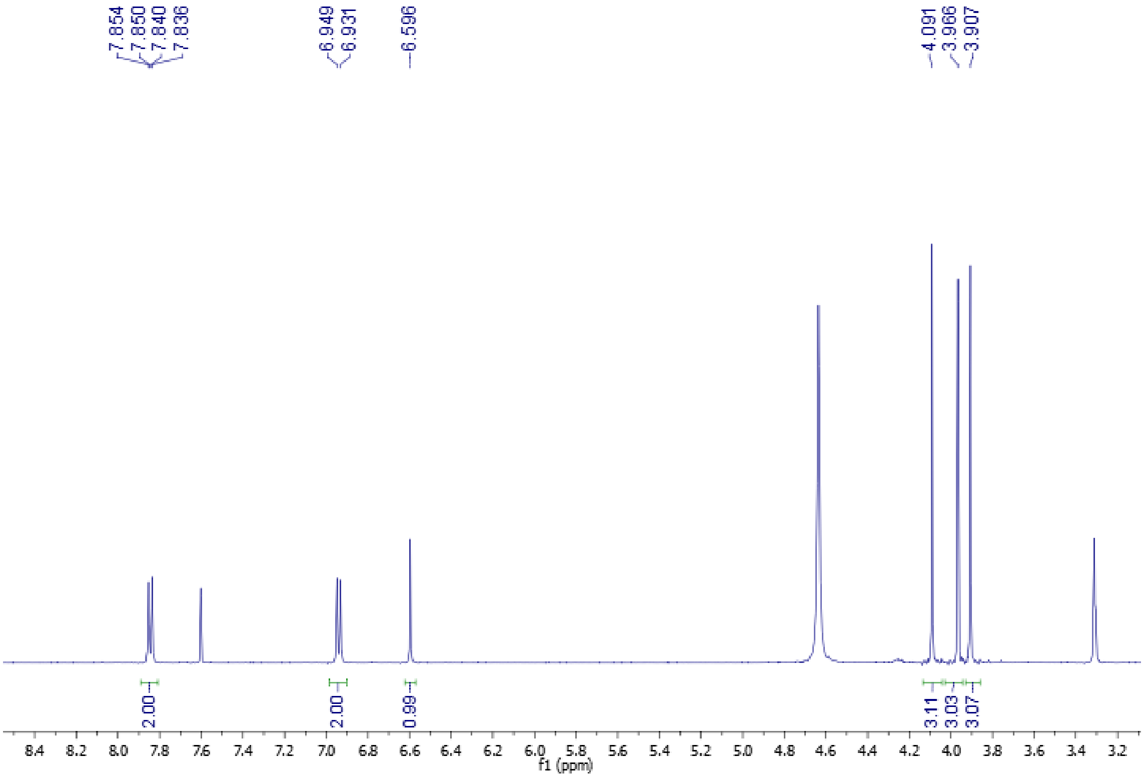


**Figure S2.** The $^{1}$H NMR spectrum of **AB1** in methanol-*d*4.

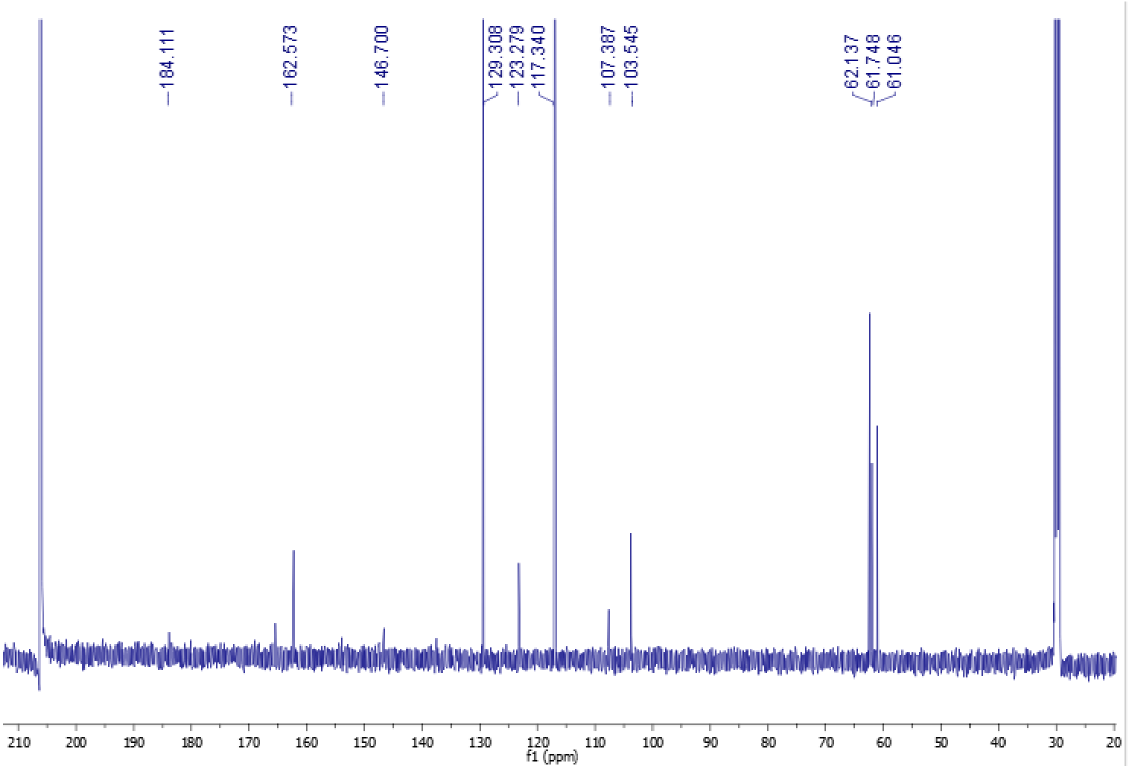


**Figure S3.** The $^{13}$C NMR spectrum of **AB1** in acetone-$d_6$.

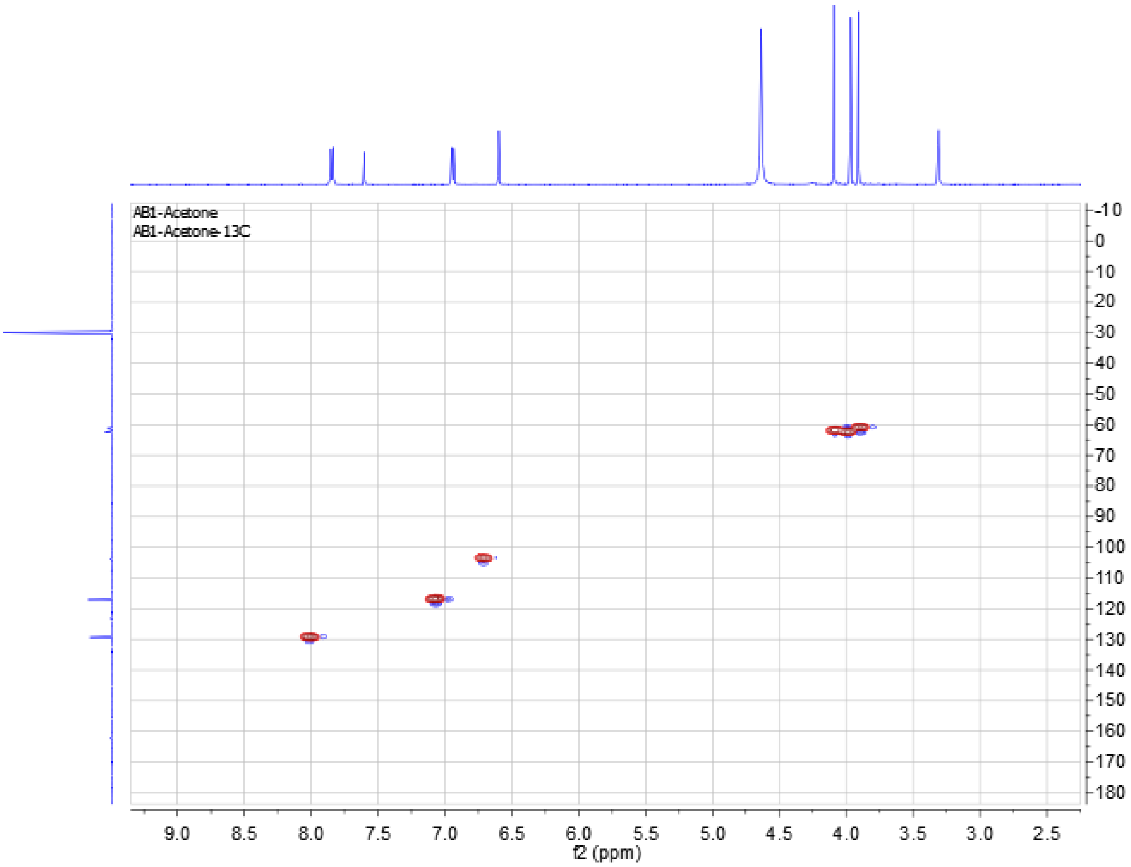


**Figure S4.** HSQC spectrum of **AB1** in acetone-$d_6$.

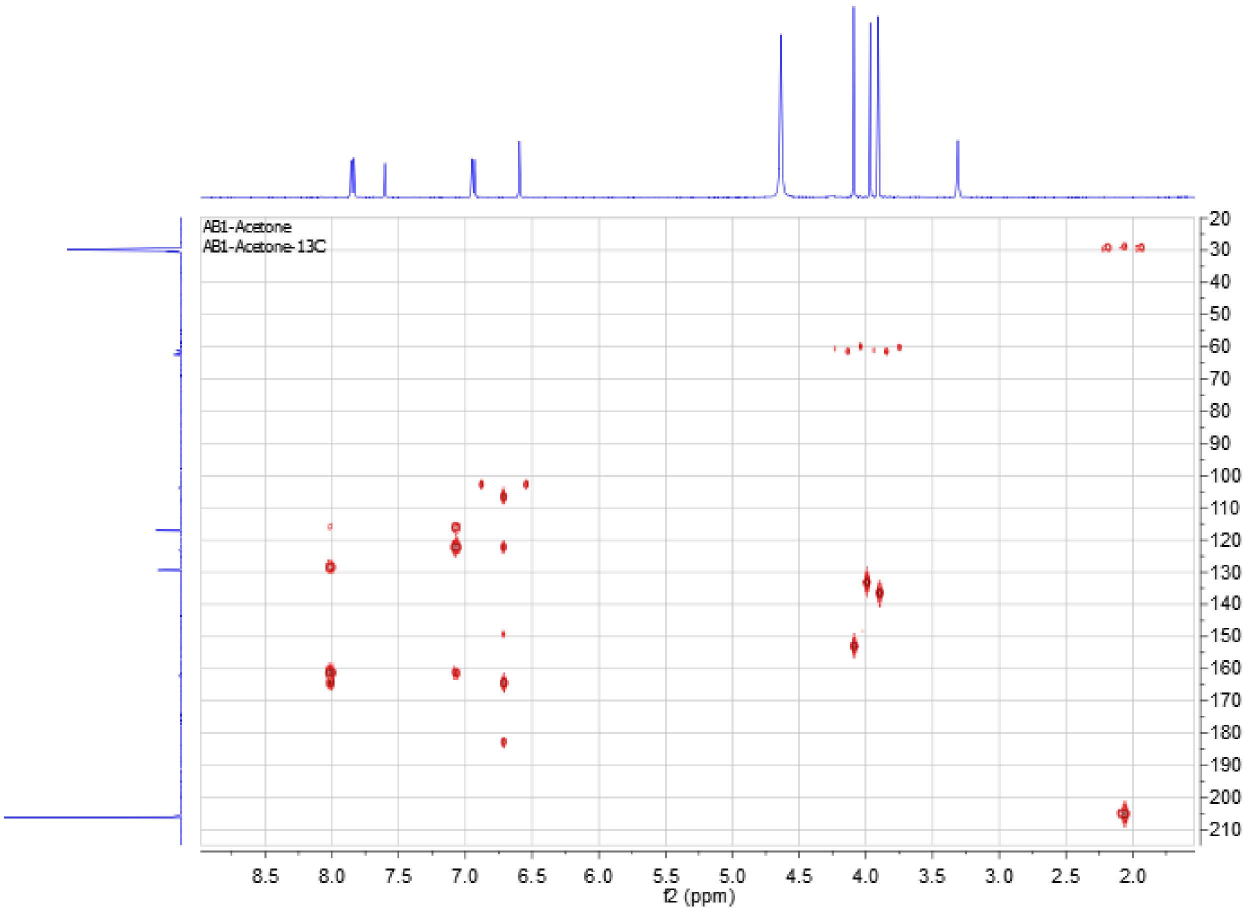


**Figure S5.** HMBC spectrum of **AB1** in Acetone-$d_6$.

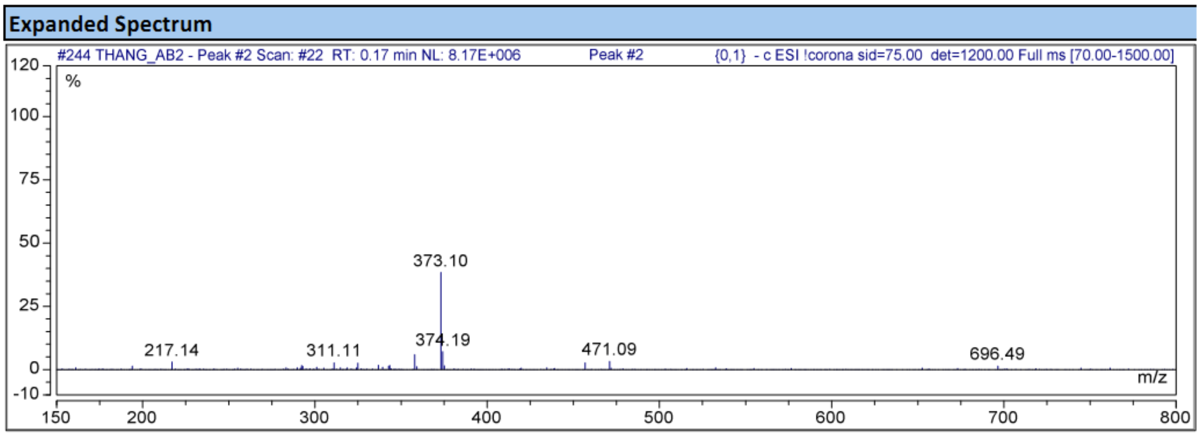


**Figure S6.** EI mass spectrum of **AB2.**

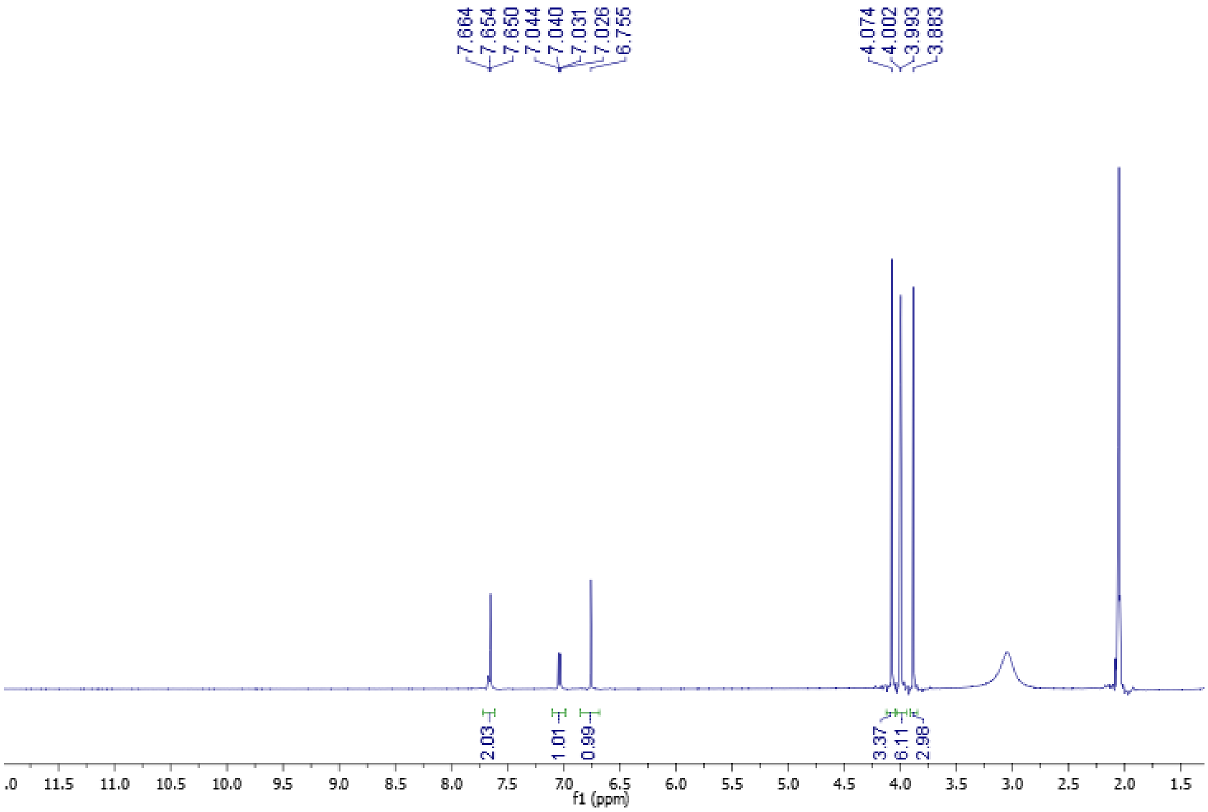


**Figure S7.** The $^{1}H$ NMR spectrum of **AB2** in acetone-$d_6$.

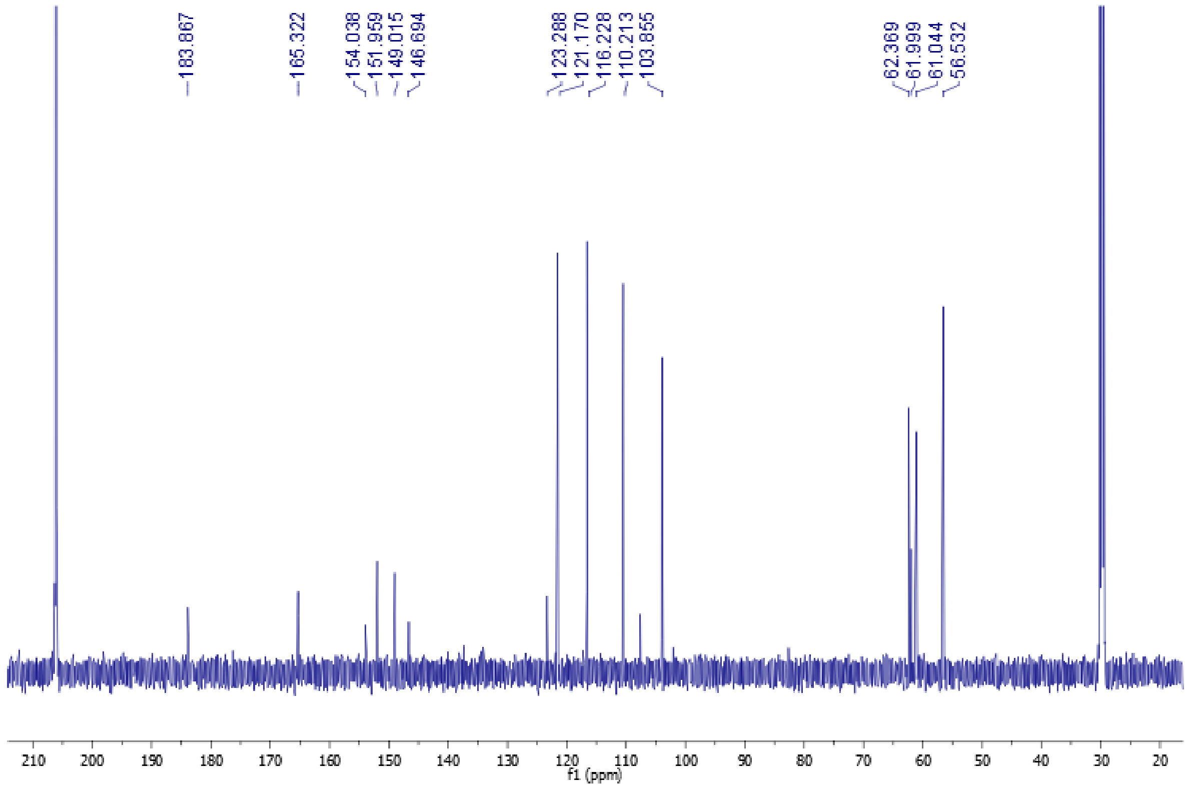


**Figure S8.** The $^{13}C$ NMR spectrum of **AB2** in acetone-$d_6$

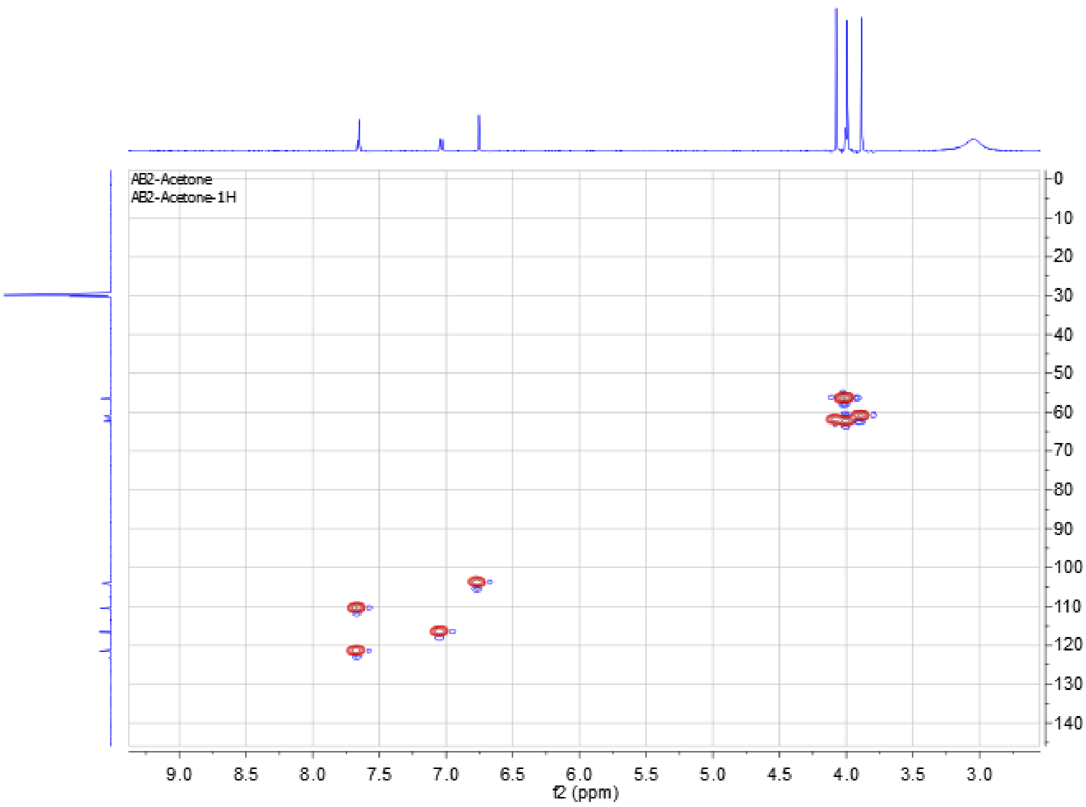


**Figure S9.** HSQC spectrum of **AB2** in acetone-$d_6$.

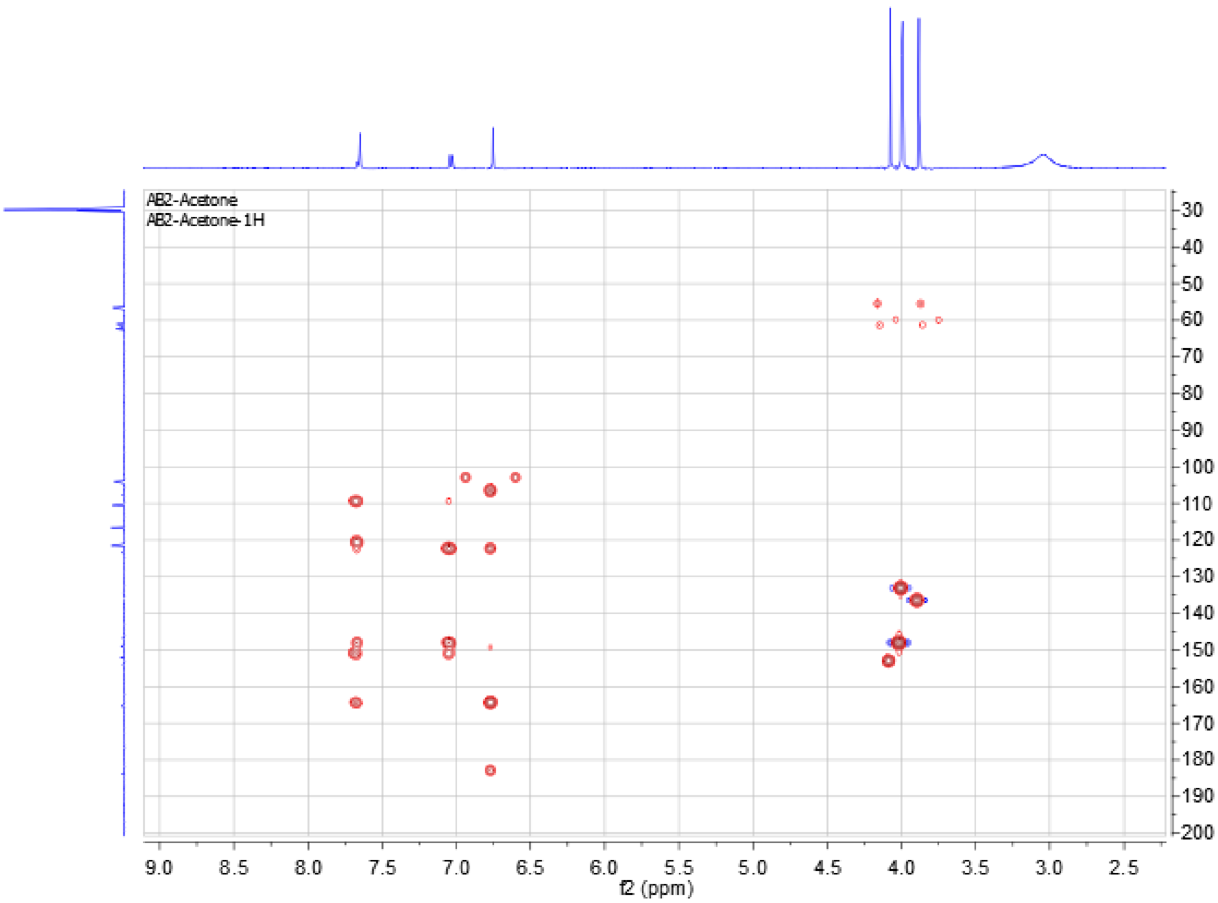

**Figure S10.** HMBC spectrum of **AB2** in Acetone-$d_6$.

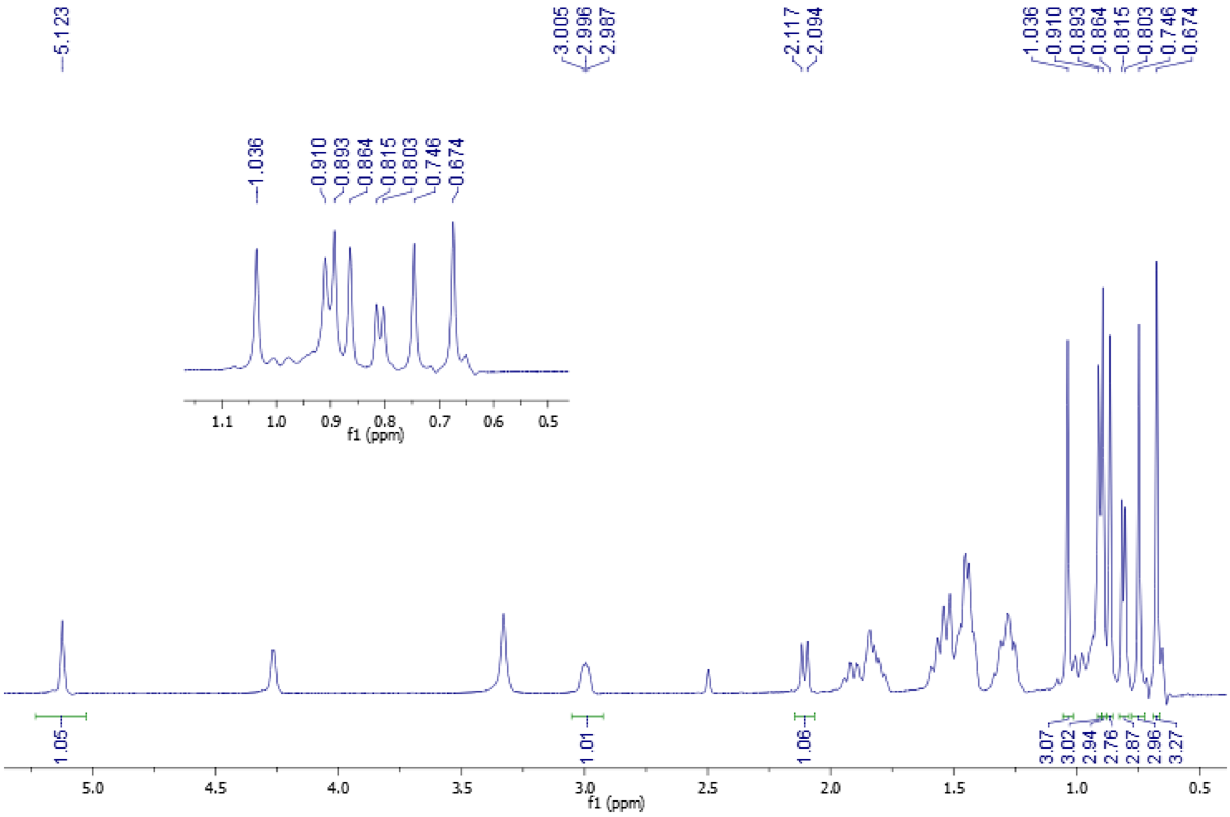


**Figure S11.** The $^{1}$H NMR spectrum of **AB3** in DMSO-$d_6$.

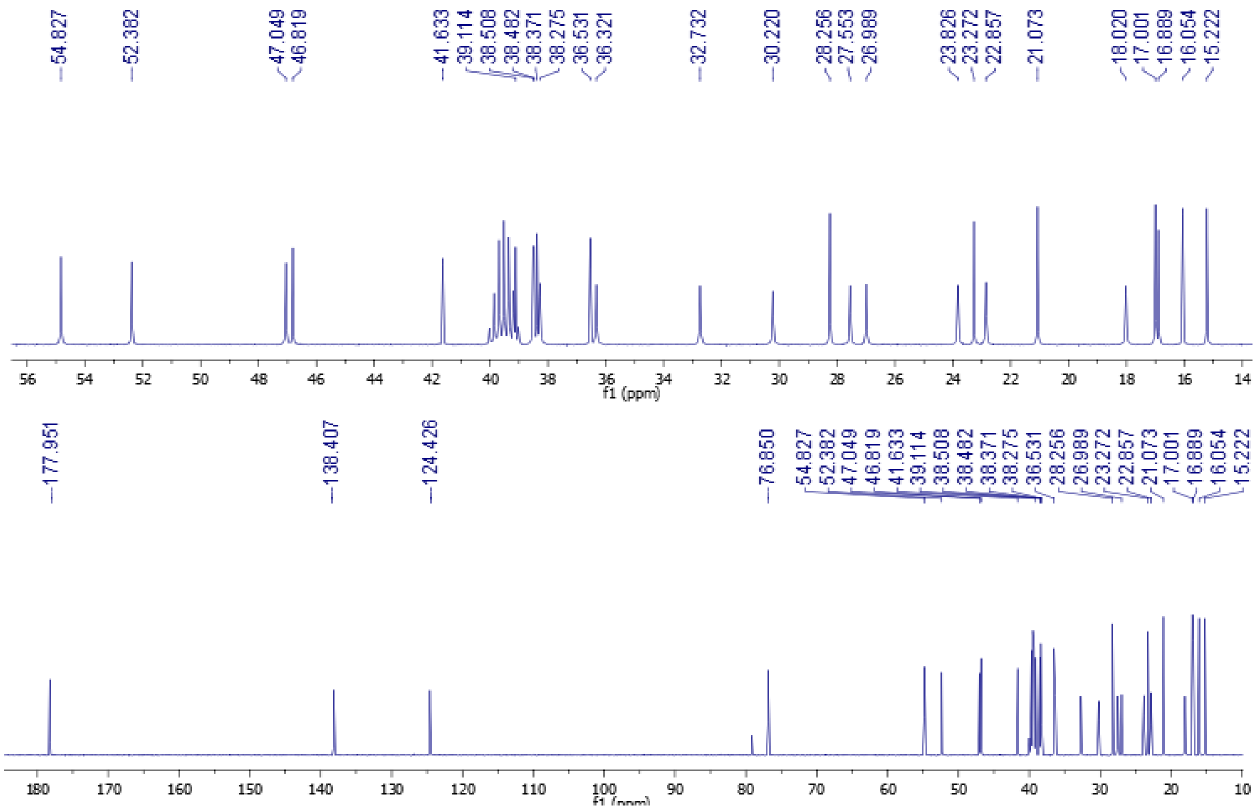


**Figure S12.** The $^{13}$C NMR spectrum of **AB3** in DMSO-*d*6.
